# Prognostic impact of nectin-like molecule-5 (CD155) expression in non-small cell lung cancer

**DOI:** 10.1186/s12967-024-05471-6

**Published:** 2024-09-12

**Authors:** Xitlally Popa-Navarro, Alejandro Avilés-Salas, Norma Hernández-Pedro, Mario Orozco-Morales, Enrique Caballé-Pérez, Cesar Castillo-Ruiz, José Lucio-Lozada, Pedro Barrios-Bernal, Juan-Manuel Hernandez-Martinez, Oscar Arrieta

**Affiliations:** 1 Thoracic Oncology Unit, Instituto Nacional de Cancerología (INCan), Mexico City, 14080 Mexico; 2 Pathology department, Instituto Nacional de Cancerología (INCan), Mexico City, 14080 Mexico; 3 Personalized Medicine Laboratory, Instituto Nacional de Cancerología (INCan), Mexico City, 14080 Mexico; 4 CONAHCYT-Instituto Nacional de Cancerología, Mexico City, Mexico

**Keywords:** CD155, Nectin-like molecule 5, Non-small-cell lung cancer, Immune checkpoints, PD-L1, Oncogene driver mutations

## Abstract

**Background:**

CD155 is a transmembrane protein that inhibits antitumor immune response and represents a predictor of worse prognosis in non-small-cell lung cancer (NSCLC). However, it remains unexplored its association with clinical characteristics and genomic status of Latin American patients. This study characterizes the CD155 expression and its clinical implications in this population.

**Methods:**

Tissue biopsies from 86 patients with locally-advanced or metastatic NSCLC were assessed for CD155 protein expression, *ALK* rearrangements and *EGFR* mutations. Cutoff values for high CD155 expression (CD155^high^) were determined from receiver operating characteristic (ROC) curves according to 2-year survival. It was evaluated its association with clinicopathological features, median progression-free survival (mPFS) and overall survival (mOS).

**Results:**

the cutoff score for CD155^high^ was 155 in the entire cohort and in patients without oncogenic alterations, and it was 110 in patients with oncogenic alterations. Eighty-four patients (97.7%) were CD155 positive, of which fifty-six (65.0%) had CD155^high^. *EGFR* L858R mutation related to lower CD155 IHC score than exon 19 deletion. Individuals with CD155^high^ showed a shorter mOS (13.0 vs. 30.8 months; HR: 1.96 [95% CI, 1.15–3.35]; *p* = 0.014). Patients without oncogenic alterations having a CD155^high^ displayed shorter mPFS (1.6 vs. 6.4 months, HR: 2.09 [95% CI, 1.06–4.20]; *p* = 0.034) and mOS (2.9 vs. 23.1 months; HR: 1.27 [95% CI, 1.07– 4.42]; *p* = 0.032). Patients with oncogenic alterations having CD155^high^ only showed a trend to shorter mOS (26.3 vs. 52.0 months; HR: 2.39 [95% CI, 0.98–5.83]; *p* = 0.058).

**Conclusion:**

CD155^high^ is a predictor of worse outcomes in patients with advanced NSCLC, predominantly among those without oncogenic alterations. CD155 could be a potential biomarker and a molecular target in patients with poor responses to current therapies.

**Supplementary Information:**

The online version contains supplementary material available at 10.1186/s12967-024-05471-6.

## Introduction

Although immune checkpoint-based therapy has marked a milestone in advanced lung cancer, only 30% of patients respond favorably to monotherapy with immune checkpoint inhibitors (ICIs) against programmed cell death ligand 1 (PD-L1) [1]. These results may derive from a poor selection of candidates for treatment, generally based on PD-L1 expression, along with the potential involvement of tumor-intrinsic mechanisms of primary resistance, such as defective antigen presentation and promotion of an immunosuppressive tumor microenvironment [2]. In this context, extensive research has focused on identifying alternative immune checkpoints of oncological importance.

Emerging evidence indicates that the Cluster of Differentiation 155 (CD155) may represent a novel immunological target. This member of the nectin-like family is upregulated in the tumor microenvironment by cellular stress, inflammatory cytokines, and cell proliferation and constitutes a relevant intermediary of adhesion, proliferation, and tumor migration [3]. In addition to these functions, CD155 also has an immunomodulatory role, promoting immune evasion by interacting with certain receptors on CD8 + T and Natural Killer (NK) cells, including T cell immunoglobulin and immunoreceptor tyrosine-based inhibitory motif domain (TIGIT) and Cluster of Differentiation 96 (CD96), DNAX accessory molecule-1 (DNAM-1) which ultimately downregulate production of interferon gamma (IFNγ) and interleukine-2 (IL-2) [4], promote the secretion of IL-10 by dendritic cells [5], and suppress cell-dependent cytotoxic responses against cancer cells [6].

In lung cancer (LC), CD155 is expressed in 38–48% of cases [1, 7] and is correlated with advanced stage, pleural/vascular invasion, PD-L1 positivity [1], higher bronchoalveolar tumor ratio [8], and shorter survival outcomes [3]. Additionally, CD155 is less frequent among never-smokers and in well-differentiated tumors [1]. Moreover, positive CD155 expression was correlated with shorter disease-free survival rates (*p* = 0.0004) in early- or advanced-stage NSCLC patients, which also represented an independent factor for unfavorable outcomes (*p* = 0.029) [8]. Similarly, worse clinical responses to anti-PD-1 therapy were found in NSCLC cases harboring CD155 positivity (ORR 25.6% vs. 54.8%; *p* < 0.01), even as first-line or later-line treatment [7]. In addition, high CD155 expression correlated with a shorter median overall survival (mOS: 16.2 vs. 29.87 months; *p* = 0.001) among other lung cancer subtypes, such as small-cell lung carcinoma (SCLC) [9]. Taken together, these findings suggest that CD155 expression is associated with survival and immunotherapy efficacy in lung cancer.

Moreover, preclinical studies have shown that CD155 may have a poorly described interplay with oncogenic driver mutations in diverse neoplasms. For instance, mutations in the Kirsten rat sarcoma viral oncogene (KRAS) have been shown to increase CD155 surface expression in colorectal cancer cells [9]. This is relevant, as CD155 has been reported to promote cell cycle progression in Ras-mutated cell lines [10]. Similarly, cell signaling derived from mesenchymal epithelial transition factor (c-MET) upregulates CD155 in medulloblastoma, thus enhancing its growth and invasiveness [11]. Despite these findings, there is limited clinical evidence regarding the prognostic role of CD155 in individuals with NSCLC harboring the most common oncogenic alterations, such as Epidermal Growth Factor Receptor (*EGFR*) or anaplastic lymphoma kinase (*ALK*) alterations. Therefore, this study comprehensively analyzed the clinical and prognostic features of CD155 in NSCLC in a Latin American population.

## Materials and methods

### Study design and selection criteria

An observational cohort study was conducted on patients with advanced lung cancer diagnosed between January and October 2019 at the Thoracic Oncology Unit of the Instituto Nacional de Cancerología (INCan). Individuals with confirmed diagnosis of lung cancer undergoing at least one line of anticancer therapy and having available histologic tumor samples were eligible for analysis. The electronic medical records of the included patients were reviewed to obtain relevant clinical and pathological data, including age, sex, smoking history, wood smoke exposure, ECOG PS status, clinical disease stage, *EGFR* and/or *ALK* mutational status, localization of metastatic disease, tumor grade, histologic subtype, and PD-L1 expression.

#### Ethical approval

The Institutional Ethics and Scientific Board Review Committee approved this study [(018/063(ICI) (CEI/1303/18)]. All personal data from enrolled patients were kept confidential using an intern number code to identify samples, and not personal data, and thereby, informed consent was not applicable.

### Immunohistochemistry procedure for CD155 and PD-L1

Tissue sections of 3 μm formalin-fixed paraffin-embedded tissue were deparaffinized and stained with hematoxylin-eosin (HE) to confirm the histopathological diagnosis. For CD155 expression, 88 biopsies were analyzed using immunohistochemistry (IHC). Briefly, slices were blocked for endogenous peroxidase activity using hydrogen peroxide. Antigen retrieval was performed using an immune heat-DNA retriever citrate (BSB 0023; BioSB, Inc.). The samples were washed with 1X Tris-buffered saline (TBS Automation Wash Buffer, 40x) and incubated with CD155 rabbit monoclonal antibody (mAb) (clone: D8A5G, 1: 50, cat#81,254; Cell Signaling Technology, USA) at room temperature for 45 min. The reaction was visualized using a MACH 4 universal horseradish peroxidase (HRP) polymer kit (M1U539, BioCare), followed by incubation with diaminobenzidine for 3 min. The sections were counterstained with hematoxylin and ammonium hydroxide. Isotype-matched IgG was used as a control for staining.

A pathologist evaluated the adequacy of the specimens for IHC analysis on the positively charged glass slides. The primary antibodies used were rabbit polyclonal antibodies against the poliovirus receptor (PVR) diluted 1:100 (Cell Signaling Technology, Massachusetts, USA, Cat. 81,254). Sections were deparaffinized in xylene, heat-treated for 20 min in 10 mM citrate-phosphate buffer (pH 6.0) for antigen retrieval, incubated with the primary antibody, and stained using the EnVision HRP Universal Kit Rabbit Mouse (DAB) (K1390; Dako, Glostrup, Denmark). We defined CD155 positivity as strong staining of 5% or more cancer cells within a tumor. Prostatic cancer and endometrial adenocarcinoma tissues were used as the positive controls for CD155 staining.

PD-L1 expression was evaluated using a primary antibody specific for PD-L1 SP263 (Roche, Basel, Switzerland) according to the manufacturer’s instructions. PD-L1 IHC assay was performed using the VENTANA PD-L1 (SP263) automated system (Roche, Basel, Switzerland). The PD-L1 tumor proportion score (TPS) was calculated as the percentage of at least 100 tumor cells with complete or partial membrane staining. PD-L1 positive samples were defined using a TPS threshold ≥ 1% [12].

### Molecular biology assessment of oncogene alterations

*ALK* gene rearrangements were assessed using the Vysis ALK Break Apart FISH Probe Kit (Abbott, Chicago, Illinois, United States; catalog number: 06N38-023) and Vysis LSI ALK Dual Color Break Apart FISH Probe (Abbott, Chicago, Illinois, United States; catalog number: 06N38-023).

DNA was extracted from paraffin-embedded tissue sections using the QIAamp DNA FFPE Tissue Kit (Qiagen, Hilden, Germany). Catalog number: 56,404). *EGFR* mutations (exons 18, 19, 20, and 21) were detected using the Therascreen RGQ PCR Kit (Qiagen, Hilden, Germany; Catalog number: 870,121) by real-time PCR using a Rotor-GeneQ 5-plex HRM (Qiagen, Hilden, Germany; Catalog number: 9,002,370) according to the manufacturer’s instructions.

### Statistical analysis

The cut-off values for defining high or low CD155 staining were estimated using receiver operating characteristic (ROC) curves according to 2-year survival for the whole population and subgroups with or without driver oncogenic alterations using GraphPad Prism 9.0.1 for macOS (Dotmatics, California, United States). Continuous variables, including age, packs per year, and PD-L1 TPS were reported as means and standard deviations (SD), or as medians and interquartile ranges (IQR) based on data distribution assessed by Kolmogorov–Smirnov Test. According to data distribution, comparisons for continuous variables between groups were evaluated using the Student’s t-test or Mann–Whitney U-test. Categorical variables, such as high or low CD155 expression and clinicopathological features were reported as frequencies and proportions, and comparisons between them were analyzed by χ2 test or Fisher exact test, based on their distribution. Survival was examined using the Kaplan–Meier method, and the significance of differences was evaluated using a log-rank test. Variable effects on survival time were investigated using the Cox regression model. Statistical significance was set at *p* < 0.05. All statistical analyses were performed using SPSS software (version 19.0; International Business Machines Corporation, Chicago, Illinois, USA).

## Results

### Clinical and histopathological characteristics

A total of 88 lung cancer samples were assessed for CD155 expression; two patients were excluded because of non-specific IHC staining (Supplementary Fig. 1). Thus, a total of 86 patients were included in survival analysis. Seventy-two patients were included in response assessment; 14 were excluded due unavailability of data. The demographic and clinicopathological characteristics of the patients are summarized in Table 1. The median age of the included individuals was 60.5 years (range, 52–72 years); most were female (60.5%), never smokers (53.5%), and non-exposed to wood smoke (62.8%). They showed a good performance status (ECOG 0–1) (82.6%) and advanced-stage disease (IV) (84.9%). The most common metastatic sites in this cohort were bone (40.7%), pleura (36.0%), contralateral lung (29.1%), and central nervous system (CNS) (25.6%). Adenocarcinoma was the predominant histological subtype (*n* = 78 [90.7%]), being the solid pattern the most frequent subtype (31.4%), followed by acinar (26.7%), papillary (5.8%), micropapillary (3.5%), and lepidic (2.3%). High-grade tumors (poorly differentiated) were identified in 42 samples (48.8%). PD-L1 positivity (TPS > 1%) was detected in 45 (52.3%) patients and high PD-L1 expression (TPS > 50%) was detected in 18 (20.9%) patients. Oncogenic *EGFR* mutations were present in 31 patients (36.0%) and predominantly represented by exon 19 deletions (27.9%), whereas *ALK* rearrangements occurred in 10 patients (11.6%). Targeted therapy and chemotherapy were used in 45.3% and 40.7% of patients, respectively. Anti-PD-(L)1 agents were administered to 13 patients (15.1%).

**Table 1:** Clinical characteristics according to mutational status and CD155 expression

Clinical characteristics Total *N* (%) 86 (100%)	Oncogene alterations					
	Negative			Positive		
	**CD155**		*P value*	**CD155**		*P value*
	**High** n (%) 28 (62.2)	**Low** n (%) 17 (37.8)		**High** n (%) 28 (58.5)	**Low** n (%) 13 (38.4)	
**Age**,** median (range)**: 60.5 (52–72)	73 (48–83)	61 (43–90)	0.141	64 (33–70)	57.50 (33–80)	0.749
**Age**,** n (%)**						
< 60: 42 (48.8)	13 (68.4)	6 (31.6)	0.463^‡^	17 (73.9)	6 (26.1)	0.382^‡^
≥ 60: 44 (51.2)	15 (57.7)	11 (42.3)		11 (61.1)	7 (38.9)	
**Sex**,** n (%)**						
Male: 34 (39.5)	13 (56.5)	10 (43.5)	0.420^‡^	8 (72.7)	3 (27.3)	0.513*
Female: 52 (60.5)	15 (68.2)	7 (31.8)		20 (66.7)	10 (33.3)	
**ECOG PS**,** n (%)**						
0–1: 71 (82.6)	24 (61.5)	15 (38.5)	0.593*	21 (65.6)	11 (34.3)	0.399*
≥ 2: 15 (17.4)	4 (66.7)	2 (33.3)		7 (77.8)	2 (22.2)	
**Smoking status**,** n (%)**						
Current or former: 40 (46.5)	18 (62.1)	11 (37.9)	0.977^‡^	8 (72.7)	3 (27.3)	0.513*
Never: 46 (53.5)	10 (62.5)	6 (37.5)		20 (66.7)	10 (33.3)	
**Pack per years**, median (range): 15.5 (7.7–40)	30 (8-105)	16 (3–80)	0.640	10 (2–27)	2.38 (0–25)	0.524
**Wood-smoke exposure**,** n (%)**						
Negative: 54 (62.8)	16 (59.3)	11 (40.7)	0.616^‡^	20 (74.1)	7 (25.9)	0.269^‡^
Positive: 32 (37.2)	12 (66.7)	6 (33.3)		8 (57.1)	6 (42.9)	
**Histology**,** n (%)**						
Adenocarcinoma: 78 (90.7)	23 (60.5)	15 (39.5)	0.462*	27 (67.5)	13 (32.5)	0.683*
Epidermoid: 08 (9.3)	5 (71.4)	2 (28.6)		1 (100)	0 (0)	
**Tumor grade**,** n (%)**						
Low: 02 (02.3)	0 (0)	1 (100)	0.376^‡^	0 (0)	1 (100)	0.116^‡^
Moderately: 33 (38.4)	9 (64.3)	5 (35.7)		11 (57.9)	8 (42.1)	
High: 42 (48.8)	15 (68.2)	7 (31.8)		16 (80)	4 (20)	
Not evaluated: 9 (10.5)						
**Adenocarcinoma subtype**,** n (%)**						
Lepidic: 02 (02.3)	0 (0)	1 (100)	0.315^‡^	0 (0)	1 (100)	**0.042** ^‡^
Acinar: 23 (26.7)	6 (60)	4 (40)		6 (46.2)	7 (53.8)	
Papillary: 05 (05.8)	3 (75)	1 (25)		1 (100)	0 (0)	
Micropapillary: 03 (03.5)	0 (0)	2 (100)		0 (0)	1 (100)	
Solid: 27 (31.4)	7 (63.6)	4 (36.4)		14 (87.5)	2 (12.5)	
Not evaluated: 26 (30.2)						
**Clinical stage**,** n (%)**						
Stage III: 13 (15.1)	6 (50)	6 (50)	0.308^‡^	1 (100)	0 (0)	0.683*
Stage IVA-IVB: 73 (84.9)	22 (66.7)	11 (33.3)		27 (67.5)	13 (32.5)	
**PD-L1 expression**,** n (%)**						
TPS < 1%: 31 (36.0)	6 (54.5)	5 (45.5)	0.534^‡^	11 (55)	9 (45)	0.051*
TPS ≥ 1%: 45 (52.3)	17 (65.4)	9 (34.6)		16 (84.2)	3 (15.8)	
Not evaluated: 10 (11.6)						
**PD-L1 expression**,** n (%)**						
TPS < 50%: 58 (67.4)	14 (58.3)	10 (41.7)	0.387*	22 (64.7)	12 (35.3)	0.140*
TPS ≥ 50%: 18 (20.9)	9 (69.2)	4 (30.8)		5 (100)	0 (0)	
Not evaluated: 10 (11.6)						
**PD-L1 TPS**, median (range): 5 (0-38.7)	5 (0–70)	22.5 (0-100)	0.926	0 (0–5)	3 (0–90)	0.103
**Metastatic sites**,** n (%)**						
Pleural effusion: 34 (39.5)	9 (69.2)	4 (30.8)	0.395*	11 (52.4)	10 (47.6)	0.027*
Lymph nodes: 10 (11.6)	5 (62.5)	3 (37.5)	0.635*	0 (0)	2 (100)	0.095*
Contralateral lung: 25 (29.1)	7 (87.5)	1 (12.5)	0.114*	12 (70.6)	5 (29.4)	0.790^‡^
Pleura: 31 (36.0)	6 (54.5)	5 (45.5)	0.482^‡^	11 (55)	9 (45)	0.073*
Central Nervous System: 22 (25.6)	8 (72.7)	3 (27.3)	0.346*	7 (63.6)	4 (36.4)	0.487*
Liver: 11 (12.8)	4 (100)	0 (0)	0.144*	4 (57.1)	3 (42.9)	0.408*
Adrenal: 13 (15.1)	5 (71.4)	2 (28.6)	0.483*	4 (66.7)	2 (33.3)	0.632*
Mediastinum: 7 (8.1)	2 (66.7)	1 (33.3)	0.698*	4 (100)	0 (0)	0.192*
Bone: 35 (40.7)	7 (50.0)	7 (50.0)	0.199^‡^	12 (57.1)	9 (42.9)	0.108*
Other: 15 (17.4)	4 (80)	1 (20)	0.381*	6 (60)	4 (40)	0.390*
**Metastatic sites**,** n (%)**						
0 sites: 10 (11.6)	4 (40)	6 (60)	0.241^‡^	0 (0)	0 (0)	0.021^‡^
1 site: 32 (37.2)	10 (66.7)	5 (33.3)		15 (88.2)	2 (11.8)	
2 sites: 21 (24.4)	9 (81.8)	2 (18.2)		6 (60)	4 (40)	
≥ 3 sites: 23 (26.7)	5 (55.6)	4 (44.4)		7 (50)	7 (50)	
**EGFR mutation**,** n (%)**						
Absent: 45 (52.3)	22 (62.9)	13 (37.1)	-	8 (80.0)	2 (20.0)	0.308*
Present: 31 (36.0)	0 (0)	0 (0)		20 (64.5)	11 (35.5)	
Not evaluated: 10 (11.6)						
**EGFR subtype**,** n (%)**						
Exon 19 deletion: 24 (27.9)	0 (0)	0 (0)	-	17 (70.8)	7 (29.2)	0.180*
Exon 21 L858R mutation: 07 (08.1)	0 (0)	0 (0)		3 (42.9)	4 (57.1)	
Not evaluated: 55 (64.0)						
**ALK alterations**,** n (%)**						
Absent: 65 (75.6)	22 (64.7)	12 (35.3)	-	20 (64.5)	11 (35.5)	0.308*
Present: 10 (11.6)	0 (0)	0 (0)		8 (80.0)	2 (20.0)	
Not evaluated: 11 (12.8)						
**First-line treatment**,** n (%)**						
Chemotherapy: 35 (40.7)	20 (58.8)	14 (41.2)	0.543^‡^	1 (100.0)	0 (0.0)	0.614^‡^
Targeted therapy: 39 (45.3)	0 (0)	0 (0)		26 (66.7)	13 (33.3)	
Surgery: 02 (2.32)	1 (50.0)	1 (50.0)		0 (0)	0 (0)	
Best Medical Support: 10 (11.6)	7 (77.8)	2 (22.2)		1 (100.0)	0 (0)	
**Immunotherapy**,** n (%)**						
Anti–PD(L)-1: 12 (13.9)	4 (44.4)	5 (55.55)	0.255^‡^	1 (33.3)	2 (66.4)	0.232*
Anti–PD(L)-1 plus CTX: 01 (01.1)	0 (0)	0 (0)		0 (0.0)	1 (100)	
**Objective response**,** n (%)**						
Absent: 41 (47.7)	16 (64.0)	9 (36.0)	0.264*	11 (68.8)	5 (31.3)	
Present: 31 (36.0)	4 (44.4)	5 (55.6)		14 (63.6)	8 (36.4)	0.743^‡^
Not evaluated: 14 (16.3)						

CD155, cluster of differentiation 155. ECOG PS, Eastern Cooperative Oncology Group Performance Status. PD-L1 TPS, Programmed Dead Ligand 1 tumor proportion score. EGFR, Epidermal Growth Factor. ALK, anaplastic lymphoma kinase.L858R, missense mutation causing an exchange of leucin for arginine in amino acid 959. Multiple metastatic sites can be found in a single patient. Objective response rate was defined as complete response or partial response according to RECIST 1.1 criteria. Statistical differences were determined by ^‡^ Chi square test and *Fisher exact test. Statistical significance was set at *p* < 0.05

### CD155 expression according to genomic and immunologic characteristics

**Fig. 1:**
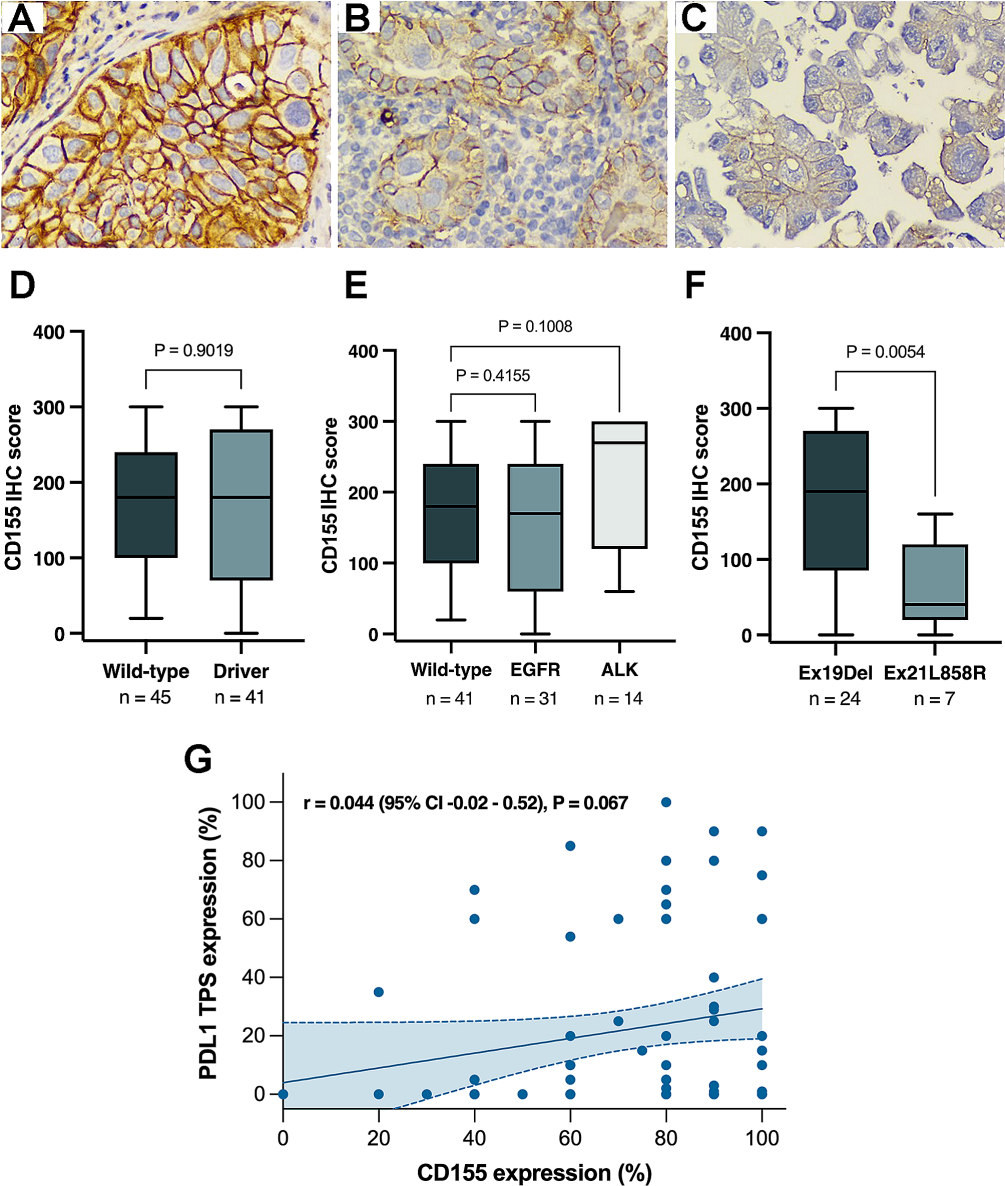
CD155 expression on tissue: high (**A**), moderate (**B**), mild (**C**). CD155 IHC score according to oncogene alterations (**D**-**F**). Correlation between PD-L1 and CD155 expressions (**G**). CD155, Cluster of Differentiation 155. IHC, immunohistochemistry. PD-L1 TPS, Programmed Dead Ligand 1 Tumor Proportion Score. EGFR, Epidermal Growth Factor. ALK, anaplastic lymphoma kinase. DelEx19, exon 19 deletion.L858R, missense mutation causing an exchange of leucin for arginine in amino acid 858. WT, wild type. Comparisons among groups were performed using U Mann Whitney test (**G**-**I**), and spearman coefficient (**J**). All images (**A**-**C**) are presented at a magnification of 400X

As shown in Fig. 1A-C, CD155 expression was observed in both the plasma membrane and the intracellular compartments. High CD155 expression (CD155^high^) was defined as H-scores over 155 for the entire cohort (*p* = 0.006) and for cases without oncogenic alterations (*p* = 0.067), and it was over 110 in patients with oncogenic alterations (*p* = 0.015) (Supplementary Fig. 1A-C). Eighty-four patients (97.7%) showed CD155 positivity, of which it was CD155^high^ in fifty-six cases (65.0%). Forty-five (52.3%) patients without oncogenic alterations showed CD155 positivity, which it was high in 28 (32.5%). Thirty-nine (45.3%) patients with oncogenic alterations showed CD155 positivity, of whose it was high in 28 (32.5%) (Supplementary Table 1). Although no differences were found between individuals with or without driver mutations (*p* = 0.901) (Fig. 1D), a trend towards higher CD155 IHC score was found in individuals with *ALK* alterations than wild-type (*p* = 0.100) (Fig. 1E). In addition, higher CD155 IHC score was observed in individuals with EGFR exon 19 deletion than in patients with exon 21 L858R mutation (*p* = 0.0054) **(**Fig. 1F**)**. There was a non-statistically significant and weak correlation between PD-L1 TPS and CD155 expression (Spearman coefficient *r* = 0.044; *p* = 0.067) (Fig. 1G).

### Clinical features associated with survival outcomes

Factors with prognostic significance are shown in Tables 2, 3 and 4. Progression-free survival (PFS) was independently associated with an Eastern Cooperative Oncology Group Performance Status ≥ 2 (ECOG ≥ 2) in the entire cohort (HR: 1.92 [95% CI, 1.03–3.57]; *p* = 0.038) and in patients without oncogenic alterations (HR: 4.56 [95% CI, 1.66–12.48]; *p* = 0.003). Meanwhile, CD155^high^ was associated with PFS only in individuals without oncogenic mutations (HR: 2.04 [95% CI, 1.03–4.02]; *p* = 0.041).

**Table 2:** Clinical characteristics associated with progression-free and overall survival in the entire cohort

Entire cohort								
Characteristics	Progression-free survival				Overall survival			
	Bivariate analysis		Multivariate analysis		Bivariate analysis		Multivariate analysis	
	HR (95% CI)	*P* value	HR (95% CI)	*P* value	HR (95% CI)	*P* value	HR (95% CI)	*P* value
**Sex**								
Male	1.06 (0.66–1.70)	0.796			1.69 (1.01–2.85)	**0.047**	1.50 (0.84–2.68)	0.169
Female								
**Age**								
< 60 years								
≥ 60 years	0.75 (0.48–1.20)	0.234			1.02 (0.61–1.72)	0.925		
**ECOG PS**								
0–1								
≥ 2	2.01 (1.09–3.73)	**0.026**	1.92 (1.03–3.57)	**0.038**	1.99 (1.05–3.79)	**0.035**	2.06 (1.08–3.93)	**0.028**
**Smoking status**								
Never								
Current/former	1.25 (0.78–1.98)	0.346			1.50 (0.89–2.51)	0.121		
**Clinical Stage**								
I-IIIB								
IV	1.28 (0.46–3.59)	0.626			1.49 (0.47–4.79)	0.469		
**Histology**								
Squamous								
Adenocarcinoma	1.48 (0.68–3.26)	0.321			1.70 (0.77–3.76)	0.187		
**PD-L1 expression**								
< 1%								
> 1%	1.00 (0.99–1.03)	0.319			1.32 (0.77–2.28)	0.305		
**CD155 IHC score**								
High	1.43 (0.89–2.29)	0.133	1.37 (1.04–3.58)	0.190	1.96 (1.15–3.35)	**0.014**	2.10 (1.15–3.50)	**0.014**
Low								

CD155, cluster of differentiation 155. ECOG PS, Eastern Cooperative Oncology Group Performance Status. PD-L1 TPS, Programmed Dead Ligand 1 tumor proportion score

**Table 3:** Clinical characteristics associated with progression-free and overall survival in individuals without driver mutations

Patients without oncogenic alterations						
Characteristics	Progression-free survival				Overall survival	
	Bivariate analysis		Multivariate analysis		Bivariate analysis	
	HR (95 CI%)	*P* value	HR (95 CI%)	*P* value	HR (95 CI%)	*P* value
**Sex**						
Male	0.81 (0.43–1.53)	0.514			1.23 (0.63–2.39)	0.552
Female						
**Age**						
< 60 years						
≥ 60 years	0.61 (0.32–1.17)	0.135			0.89 (0.46–1.37)	0.730
**ECOG PS**						
0–1						
≥ 2	4.79 (1.74–13.21)	**0.002**	4.56 (1.66–12.48)	**0.003**	1.88 (0.71–4.94)	0.200
**Clinical Stage**						
I-IIIB						
IV	1.64 (0.48–5.50)	0.425			2.25 (0.67–7.54)	0.189
**Smoking status**						
Never						
Current/former	0.78 (0.40–1.50)	0.468			1.08 (0.54–2.17)	0.820
**Histology**						
Squamous						
Adenocarcinoma	1.12 (0.47–2.71)	0.189			0.94 (0.39–2.28)	0.898
**PD-L1 expression**						
< 1%						
≥ 1%	1.28 (0.61–2.69)	0.516			1.05 (0.48–2.25)	0.903
**CD155 IHC score**						
High	2.09 (1.06–4.12)	**0.034**	2.04 (1.03–4.02)	**0.041**	2.17 (1.07–4.42)	**0.032**
Low						

CD155, cluster of differentiation 155. IHC, immunohistochemistry. ECOG PS, Eastern Cooperative Oncology Group Performance Status. PD-L1 TPS, Programmed Dead Ligand 1 tumor proportion score. Multivariate analysis for overall survival was not possible to be performed, as only 1 variable was significant in bivariate analysis

**Table 4:** Clinical characteristics associated with progression-free and overall survival in individuals with driver mutations

Patients with oncogene alterations						
Characteristics	Progression-free survival		Overall survival			
	Bivariate analysis		Bivariate analysis		Multivariate analysis	
	HR (95% CI)	*P* value	HR (95% CI)	*P* value	HR (95% CI)	*P* value
**Sex**						
Male	1.39 **(**0.66–2.93**)**	0.387	1.92 **(**0.83–4.46**)**	0.129		
Female						
**Age**						
< 60 years						
≥ 60 years	0.73 **(**0.37–1.44**)**	0.361	0.96 **(**0.43–2.20**)**	0.935		
**ECOG PS**						
0–1						
≥ 2	2.02 **(**0.85–4.83**)**	0.135	3.65 **(**1.42–9.35**)**	**0.007**	3.45 **(**0.95–12.56**)**	0.060
**Smoking status**						
Never						
Current/former	1.61 **(**0.76–3.40**)**	0.210	1.19 **(**0.49–2.89**)**	0.688		
**Adenocarcinoma subtype**						
Acinar/lepidic						
Solid	1.29 **(**0.61–2.73**)**	0.512	2.56 **(**1.03–6.35**)**	**0.043**	1.62 **(**0.57–4.65**)**	0.367
**Driver mutation**						
Absent						
Present	1.19 **(**0.54–2.67**)**	0.666	1.38 **(**0.54–3.56**)**	0.494		
**Type of EGFR mutation**						
L858R						
DelEx19	1.25 **(**0.51–2.98**)**	0.630	0.77 **(**0.25–2.35**)**	0.647		
**PD-L1 expression status**						
< 1%						
≥ 1%	0.92 **(**0.46–1.83**)**	0.814	1.01 **(**0.99–1.02**)**	0.373		
**CD155 IHC score**						
High	1.20 **(**0.60–2.40**)**	0.607	2.39 **(**0.98–5.83**)**	0.056	1.64 **(**0.55–4.88**)**	0.364
Low						

CD155, cluster of differentiation 155. IHC, immunohistochemistry. ECOG PS, Eastern Cooperative Oncology Group Performance Status. PD-L1 TPS, Programmed Dead Ligand 1 tumor proportion score. EGFR, Epidermal Growth Factor. ALK, anaplastic lymphoma kinase. DelEx19, exon 19 deletion. L858R, missense mutation causing an exchange of leucin for arginine in amino acid 858. CNS, Central Nervous System. LEP, lepidic. ACN, Acinar. PAP, papillary. MCP, Micropapillary. SOL, solid. PFS was calculated from diagnosis to progression to first-line treatment. OS was determined by the period between diagnosis and death for any cause. Multivariate analysis for progression-free survival was not possible to be performed as none of the variables were significant during bivariate analysis

In addition, ECOG ≥ 2 was a predictor of overall survival (OS) in the whole cohort (HR: 2.06 [95% CI, 1.08–3.93]; *p* = 0.028) and in patients with oncogenic alterations (HR: 3.65 [95% CI, 1.42–9.35]; *p* = 0.007). Meanwhile, CD155^high^ represented a prognostic factor for OS in the entire cohort (HR: 2.10 [95% CI, 1.15–3.50]; *p* = 0.014) and in patients without oncogenic alterations (HR: 2.17 [95% CI, 1.07–4.42]; *p* = 0.032). Differently, solid adenocarcinoma pattern only represented a prognostic factor for OS in subjects having oncogenic alterations (HR: 2.56 [95% CI, 1.03–6.35]; *p* = 0.043).

### CD155 ^high^ as a predictor of survival according to genomic status

**Fig. 2:**
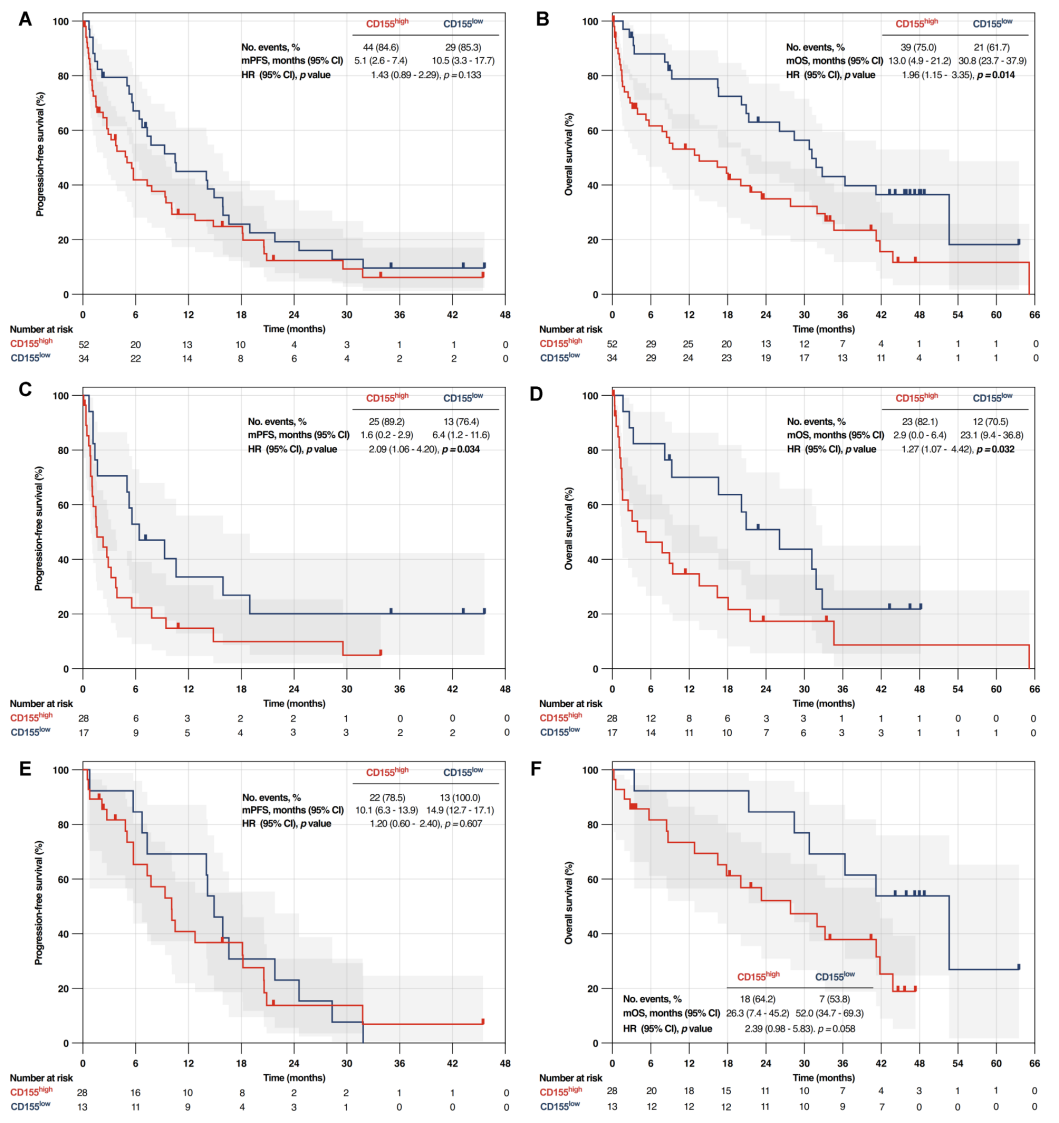
Prognostic significance of high CD155 expression on progression-free survival and overall survival of the whole cohort (**A-B**), patients without oncogene alterations (**C-D**) and individuals with oncogenic alterations (**E-F**). CD155, Cluster of Differentiation 155. HR, hazard ratio. mOS, median overall survival. mPFS, median progression-free survival. CI, confidence interval. PFS was calculated from diagnosis to progression to first-line treatment. OS was determined by the period between diagnosis and death for any cause. Log-rank test was performed to determine statistical differences between Kaplan-Meyer curves as always as p values were less than 0.05

In the entire cohort, CD155^High^ group displayed a trend to shorter median PFS (mPFS: 5.1 vs. 10.5 months; HR: 1.43 [95% CI, 0.89–2.29]; *p* = 0.133) and a significant diminish of median OS (13.0 vs. 30.8 months; HR: 1.96 [95% CI, 1.15–3.35]; *p* = 0.014) (Fig. 2A-B and Supplementary Table 2). In patients without genomic alterations, CD155^High^ group showed shorter mPFS (1.6 vs. 6.4 months, HR: 2.09 [95% CI, 1.06–4.20]; *p* = 0.034) and median OS (2.9 vs. 23.1 months; HR: 1.27 [95% CI, 1.07– 4.42]; *p* = 0.032) (Fig. 2C-D and Supplementary Table 3). In patients having oncogenic alterations, CD155^High^ group showed a trend towards shorter mOS (26.3 vs. 52.0 months; HR: 2.39 [95% CI, 0.98–5.83]; *p* = 0.058) (Fig. 2F and Supplementary Table 4).

### CD155 and PD-L1 as predictors of survival in the entire cohort

It was performed a prognostic assessment to elucidate whether CD155 and PD-L1 may act as combined prognostic factors. In terms of mPFS, no significant differences were found in patients with either high or low CD155 and/or PD-L1. However, longer mOS was identified in patients having a CD155^high^/PD-L1^low^ (20.1 months [95% CI, 7.4–32.7], *p* = 0.033) or CD155^low^/PD-L1^low^ (31.2 months [95% CI, 25.9–36.4], *p* = 0.033), and worse OS was displayed by individuals with CD155^high^/PD-L1^high^ (5.3 months [95% CI, 1.4–9.1], *p* = 0.033) or CD155^low^/PD-L1^high^ (8.7 months [95% CI, 7.6–9.8], *p* = 0.033) (Supplementary Fig. 3).

### Therapeutic response according to CD155 expression

In the entire cohort, 31 patients (36.0%) showed objective response rate (ORR) (Table 1). No significant differences were identified in terms of therapeutic response according to CD155 expression. Nonetheless, a trend to lower ORR to chemotherapy was observed in patients with CD155^high^ (20% vs. 37.5%, *p* = 0.307). No important differences were found in patients undergoing targeted therapies for EGFR mutations or ALK rearrangements (Supplementary Fig. 4).

## Discussion

This cohort study found that CD155^high^ represents a valuable prognostic factor, prominently in patients without oncogene alterations. The lack of correlation of CD155 with clinical response in patients undergoing EGFR targeted therapy implies that it may not be an appropriate predictor of benefit from tyrosine kinase inhibitors (TKIs).

Poor survival outcomes were correlated with CD155 expression in this population, particularly in chemotherapy-treated patients without oncogenic alterations, likely related to its biological role in promoting cell adhesion, motility, proliferation, survival [5], and angiogenesis [13] by Ras-Raf-MEK-ERK signaling, p27 downregulation, and upregulation of cyclin D2, cyclin E [14] and Bcl-2 protein [15]. As well, some chemotherapeutic agents are reported to upregulate CD155 expression through ATM/ATR-related kinases in breast cancer [15], probably enhancing its poor prognostic role. Furthermore, solid histological pattern is associated with CD155 in lung cancer, which predicts unfavorable survival outcomes compared to other histologic subtypes in early-staged lung cancer [16]. Finally, increased tumoral metabolic activity driven by *EGFR* alterations may also drive poor prognosis in patients with CD155 expression by enhancing glucose uptake and lipid synthesis, likely promoting tumor progression [17]; however, functional interplay between these oncogenes and CD155 remains unexplored.

This study identified trend to worse objective response rate in chemotherapy-treated patients having a CD155^High^, which is consistent with previous evidence. This has been attributed to its relationship with a solid histologic pattern, which is commonly considered a predictor for poor chemotherapy response [16]. Although previous evidence relates CD155 with lower response to EGFR TKIs in early-stage lung cancer [16], this study did not show important differences in our population. This discrepancy may mostly derive from disease stage and type of TKIs evaluated.

Like our results in patients without oncogene mutations, CD155 overexpression has demonstrated deleterious prognostic significance in multiple other tumors, including pancreatic cancer, cholangiocarcinoma, sarcoma, melanoma, breast cancer, cervical cancer, head and neck squamous cell carcinoma (HNSCC), and osteosarcoma [3, 6, 18, 19], among which, it is significantly correlated with tumor stage, lymph node, and distant metastases [3], thus suggesting that CD155 may function as poor predictors of survival regardless of cancer type.

Other nectin-like molecules have also demonstrated clinical importance in lung cancer, such as CD112 and CD113, but CD155 raises as the most representative due to its higher affinity for TIGIT, which increases its importance as a potential target of therapeutic inhibition [20]. CD113 is also frequently found in lung adenocarcinoma (25%) and only represents a deleterious prognostic factor when not co-localized with E-cadherin in cell membrane, likely allowing its binding with nectin-5 or TIGIT to promote cancer progression [21]. Otherwise, high serum levels of CD112 correlated with clinical stage, tumor size and metastatic status, but was not considered a significant predictor of progression-free survival in lung cancer [22], probably derived from its weak interaction with TIGIT [23].

Moreover, PD-L1 expression was also assessed in our population (52.3%), and its prevalence was like that in the Asian population (48.2%) [22], and higher than that in Europeans (32.6%) [23]. Although CD155 was not significantly correlated with PD-L1 expression in the entire population, CD155^high^ was associated with higher PD-L1 positivity in the oncogene-mutated subsets (*ALK* and *EGFR* alterations), which is concordant with studies in early-stage lung cancer [1]. This lack of correlation between CD155 and PD-L1 may be influenced by oncogenic mutations, since *EGFR* [24] and *ALK* [25] impair IFN-y signaling and downregulate PD-L1 expression. Despite the prognostic role of CD155 alone, PD-L1^Low^ always correlated with better PFS and OS regardless CD155 expression, which further supports their lack of correlation. Despite their known association with poor prognosis as combined factors (CD155^high^/PD-L1^high^) [26], this discrepancy might be given by their study in early-stage diseases non-including patients with oncogenic alterations.

As well, individuals with *EGFR* alterations may alternate the expression of CD155 and PD-L1 depending on their *EGFR* subtype since this study showed that individuals with exon 19 deletions mutations showed a higher CD155 expression than L858R mutations, which is complemented by previous evidence standing that L858R mutation is related to a higher PD-L1 expression than exon 19 deletions [27]. This hypothesis remains to be further confirmed, but it suggests that CD155 axis might be a valuable target in subjects with oncogene alterations conditioning low PD-L1 expression, currently classified them as not candidates for immunotherapy alone (Supplementary Fig. 5).

Current therapeutic approaches targeting CD155 axis aim to block TIGIT, indirectly promoting CD226 activity to reactivate cytotoxic immune response, upregulate IFNγ signaling and suppress IL-10 secretion by dendritic cells [23]. For example, the CITYSCAPE Fase II trial showed that tiragolumab (anti-TIGIT) plus atezolizumab (anti-PD-L1) improved response rates and survival outcomes in patients with lung cancer [28]. Although anti-TIGIT agents only improve clinical outcomes in combination with anti-PD-L1 blockers, their concomitant use constitutes a promising alternative in individuals with low PD-L1 expression [29].

The most representative limitations of this study are its retrospective nature and single institution performance. As well, histological types other than adenocarcinoma and *ALK* rearrangements were infrequent in the included population. Finally, TIGIT, CD96, and CD226 were not measured, hindering their relationship with CD155 expression, but future approaches will address this limitation. As well, only a small number of patients received immunotherapy, which limits the sub-analysis of their clinical outcomes. Finally, potential confounders of this study are multiple treatment modalities with considerably different impact on survival outcomes and inter-individual variability due to our small-sample size.

## Conclusions

A considerable proportion of patients with NSCLC harbor CD155 expression in our population. Its high expression predicts worse survival outcomes, with prominent impact on individuals without oncogene alterations. The study of the CD155 axis may promote the future development of a new generation of ICIs, of importance in poorly responsive patients to current therapeutic approaches.

## Electronic supplementary material

Below is the link to the electronic supplementary material.

Supplementary Material 1

Supplementary Material 2

Supplementary Material 3

Supplementary Material 4

Supplementary Material 5

## Data Availability

Not applicable.
