# Correction: Prognostic impact of nectin-like molecule-5 (CD155) expression in non-small cell lung cancer

**DOI:** 10.1186/s12967-025-06781-z

**Published:** 2025-07-03

**Authors:** Xitlally Popa-Navarro, Alejandro Avilés-Salas, Norma Hernández-Pedro, Mario Orozco-Morales, Enrique Caballé-Pérez, Cesar Castillo-Ruiz, José Lucio-Lozada, Pedro Barrios-Bernal, Juan Manuel Hernández-Martínez, Oscar Arrieta

**Affiliations:** 1 Thoracic Oncology Unit, Instituto Nacional de Cancerología (INCan), Mexico City, 14080 Mexico; 2 Pathology department, Instituto Nacional de Cancerología (INCan), Mexico City, 14080 Mexico; 3 Personalized Medicine Laboratory, Instituto Nacional de Cancerología (INCan), Mexico City, 14080 Mexico; 4 CONAHCYT-Instituto Nacional de Cancerología, Mexico City, Mexico

**Correction: J Transl Med 22**, **841 (2024).**

10.1186/s12967-024-05471-6

Following publication of the original article [1], the authors reported an error in the authorship. The Autor and Family names appeared reversed.

The corrected names provided in this Correction are accurate.

The original article [1] has been updated.

## References

[1] Popa-Navarro X, Avilés-Salas A, Hernández-Pedro N, et al. Prognostic impact of nectin-like molecule-5 (CD155) expression in non-small cell lung cancer. J Transl Med. 2024;22:841. 10.1186/s12967-024-05471-6 39267111 10.1186/s12967-024-05471-6 PMC11391680

