# Supplementary material for: Prognostic impact of nectin-like molecule-5 (CD155) expression in non-small cell lung cancer
Source: J Transl Med. 2024 Sep 12;22:841. doi: 10.1186/s12967-024-05471-6 (PMC11391680; doi:10.1186/s12967-024-05471-6)
Supplement: Supplementary file 1 — Supplementary Material 1 [file 12967_2024_5471_MOESM1_ESM.docx]

Mexico City, June 05^th^, 2024

**Francesco Marincola, MD**

Editor-in-Chief

Journal of Translational Medicine

Please find enclosed the version of our manuscript entitled “**Prognostic impact of nectin-like molecule-5 (CD155) expression in Non-Small Cell Lung Cancer**” by  Xitlally Popa-Navarro, Alejandro Avilés-Salas, Norma Hernández-Pedro, Mario Orozco-Morales, Enrique Caballé-Pérez, Cesar Castillo-Ruiz, José Lucio-Lozada, Pedro Barrios-Bernal, Juan-Manuel Hernandez-Martinez, Oscar Arrieta, for publication as an original scientific article in your prestigious journal.

This study (manuscript word count: 3, 127) has not been submitted or published in any other journal, and it is not under review for publication elsewhere. All authors have read and approved the manuscript and concur with the submission.

Also, Oscar Arrieta reports receiving personal fees from Pfizer, Lilly, Merck, and Bristol-Myers Squibb and grants and personal fees from AstraZeneca, Boehringer Ingelheim, and Roche, outside of this submitted work. The rest of authors declare no affiliations with or involvement in any organization or entity with any financial interest in the subject matter or materials discussed in this manuscript.

Lung cancer is the main cause of death related to oncologic disease, of which adenocarcinoma subtype is the most frequent histologic subtype. Heterogeneous response rates have been reported for immunotherapy use, likely derived from inaccurate selection methods based in PD-L1 expression. Therefore, research in novel immunologic biomarkers has pointed out that CD155 represent a valuable target for the potential development of innovative immune checkpoint inhibitors, but its study in patients with oncogene-mutated NSCLC remains unexplored. Thus, this article describes the clinical and prognostic features of CD155 expression in a Latin American population. This study demonstrated that high CD155 expression (CD155^high^) represented a predictor of worse survival outcomes in patients with advanced NSCLC, predominantly among those without oncogenic alterations. As well, it was associated with some genomic particularities; for example, it was observed a higher CD155 IHC score in patients with EGFR exon 19 deletion than L858R mutation. Moreover, CD155^high^ was associated with higher PD-L1 positivity (TPS >1%) among individuals with ALK or EGFR alterations. The study of the CD155 axis may promote the future development of a new generation of ICIs of importance in poorly responsive patients to current therapeutic approaches.

This data fills an important knowledge need in field of novel immune checkpoints, describing its expression along different clinical and genomic characteristics in our population different from most of the reported worldwide.

Kind regards,

**Oscar Arrieta, MD**

Thoracic Oncology Unit and Experimental Oncology Laboratory

Instituto Nacional de Cancerología de México (INCan)

San Fernando #22, Col. Sección XVI, Tlalpan, 14080 México, D.F., México Phone: (+52) (55) 5628-0400, ext. 832; Fax (+52) (55) 551315-1223; email: [ogar@unam.mx](mailto:ogar@unam.mx)
