# Supplementary material for: Prognostic impact of nectin-like molecule-5 (CD155) expression in non-small cell lung cancer
Source: J Transl Med. 2024 Sep 12;22:841. doi: 10.1186/s12967-024-05471-6 (PMC11391680; doi:10.1186/s12967-024-05471-6)
Supplement: Supplementary file 2 — Supplementary Material 2 [file 12967_2024_5471_MOESM2_ESM.docx]

LETTER OF RESPONSE TO REVIEWERS

REVIEWER #2

**1.1 “Table 1 is very difficult to read and interpret. I suggest intercalating lines in gray and white, as in supplementary table 1.”**

**Response**: gray and white lines were intercalated in all rows of Table 1 to enhance its interpretation and visualization.

**1.2. “N (%), driver, no driver mutations, CD155low and high, does not sum up 86 patients.”**

**Response**: This error was corrected. All data in the tables were correct, but there was an error in the sum up of total values.

**1.3. “Acronyms must be spelled out in the legend along with statistical tests.”**

**Response**: Acronyms in Table 1 were spelled out in the legend along with statistical tests as follows:

“Table 1. Clinical characteristics according to mutational status and CD155 expression. CD155, Cluster of Differentiation 155. ECOG PS, Eastern Cooperative Oncology Group Performance Status. PD-L1 TPS, Programmed Dead Ligand 1 Tumor Proportion Score. EGFR, Epidermal Growth Factor. ALK, anaplastic lymphoma kinase.L858R, missense mutation causing an exchange of leucin for arginine in amino acid 959. Statistical differences were determined by ‡ Chi square test and *Fisher exact test. Statistical significance was set at p<0.05.”

**2. “Sup Table 1. Legend must go below the table.”**

**Response**: the legend was placed below the table.

**3. Figure 1. It lacks information in the result to interpret the data. There is no legend to guide what the figure is showing.**

**Response**: The legend is placed below the figure 1, as shown:

“Figure 1. CD155 expression on tissue: high (A), moderate (B), mild (C). CD155 IHC score according to oncogene alterations (D-F). Correlation between PD-L1 and CD155 expressions (G). CD155, Cluster of Differentiation 155. IHC, immunohistochemistry. PD-L1 TPS, Programmed Dead Ligand 1 Tumor Proportion Score. EGFR, Epidermal Growth Factor. ALK, anaplastic lymphoma kinase. DelEx19, exon 19 deletion.L858R, missense mutation causing an exchange of leucin for arginine in amino acid 858. WT, wild type. Comparisons among groups were performed using U Mann Whitney test (G-I), and spearman coefficient (J). All images (A-C) are presented at a magnification of 400X.”

**4. The box plots should be replaced by bars and symbols representing individuals. With this representation it is easier for the reader to judge the author's interpretations. Such as "Although no differences were found between individuals with or without driver mutations (p=0.901) (Figure 1D), a trend towards higher CD155 expression was found in individuals with ALK alterations than in those without driver mutations (p=0.100) (Figure 1E)". As it is, the groups are similar.**

**Response:** we consider that box plots are the most appropriate graphical representation of our results, since they accurately present CD155 expression along our small-sampled study, which has already described as one of its most important limitations.

**5. “Do not agree with the statement: "There was a trend towards a statistically significant correlation between PD-L1 TPS and CD155 expression (Spearman coefficient r = 0.044; p=0.067) (Figure 1G)." If the authors want to keep the correlation graph, they should say the correlation is weak and not significant statistically.”**

**Response:** this correlation was described as non-significant in results section, as shown (**page 5, lines 225-226**):

**“**There was a non-statistically significant and weak correlation between PD-L1 TPS and CD155 expression (Spearman coefficient r = 0.044; *p*=0.067) (**Figure 1G**).**”**

**6. “I could not find the Figure legends.”**

**Response**: Figure legends are now located after references section (**page 24, lines 526-531**).

**7. “Tables 2-4 must be carefully reconstructed. It is not easy to read the data. There are a lot of blank spaces. Legend is not complete. What exactly Events, n means? e.g., Sex (Male), but below this parameter there are male, female and in each number/number. Usually, we would have a number of male/total. The same happens with the other parameters.”**

**Response**: all tables were redesigned to eliminate unnecessary blank spaces, group and homogenize headings. All median PFS and OS were relocated to supplementary section, as Cox regression models are more than enough to describe the statistical impact of clinical characteristics over survival outcomes according to the study’s objectives. As well, “Events, n” represents, in the case of progression-free survival, the number of patients showing progression of the disease regarding all patients in each subgroup, and in terms of overall survival, the number of patients unfortunately dying during follow time regarding the totality of individuals in each subgroup. “Sex (Male)” denotes the variable to which hazard ratio (HR) belongs, but as each HR is located next to their corresponding variable, we decided to eliminate these parentheses to enhance the simplicity of our tables.

**8. “I would remove the multivariate analysis from tables 2-4 and build another table including the data from the three tables.”**

**Response**: Tables 2-4 were simplified by eliminating blank columns and grouping headings as much as possible. Nonetheless, grouping all multivariate analysis in 1 table is not possible, as Cox regression in each subgroup has slight differences in their included variables. Additionally, from our perspective, placing bivariate analysis next to multivariate allows a to see which variables were significant for both, enhancing their interpretation.

**9. “Since the CD155 and PD-1L did not correlate and both are good markers for disease progression, an analysis that considers both would improve the results.”**

**Response:** this analysis was performed, and its results are described in section 3.5 as follows (**page 11, lines 251-257**):

“It was performed a prognostic assessment to elucidate whether CD155 and PD-L1 may act as combined prognostic factors. In terms of mPFS, no significant differences were found in patients with either high or low CD155 and/or PD-L1. However, longer mOS was identified in patients having a CD155high/PD-L1low (20.1 months [95% CI, 7.4-32.7], p=0.033) or CD155low/PD-L1low (31.2 months [95% CI, 25.9-36.4], p=0.033), and worse OS was displayed by individuals with CD155high/PD-L1high (5.3 months [95% CI, 1.4-9.1], p=0.033) or CD155low/PD-L1high (8.7 months [95% CI, 7.6-9.8], p=0.033) (**Supplementary Figure 3**).”

And they were presented in discussion section (**page 13, lines 308-312**):

“Despite the prognostic role of CD155 alone, PD-L1Low always correlated with better PFS and OS regardless CD155 expression, which further supports their lack of correlation. Despite their known association with poor prognosis as combined factors (CD155high/PD-L1high) [27], this discrepancy might be given by their study in early-stage diseases non-including patients with oncogenic alterations.”

Their associated Figure was located as **Supplementary Figure 3**:


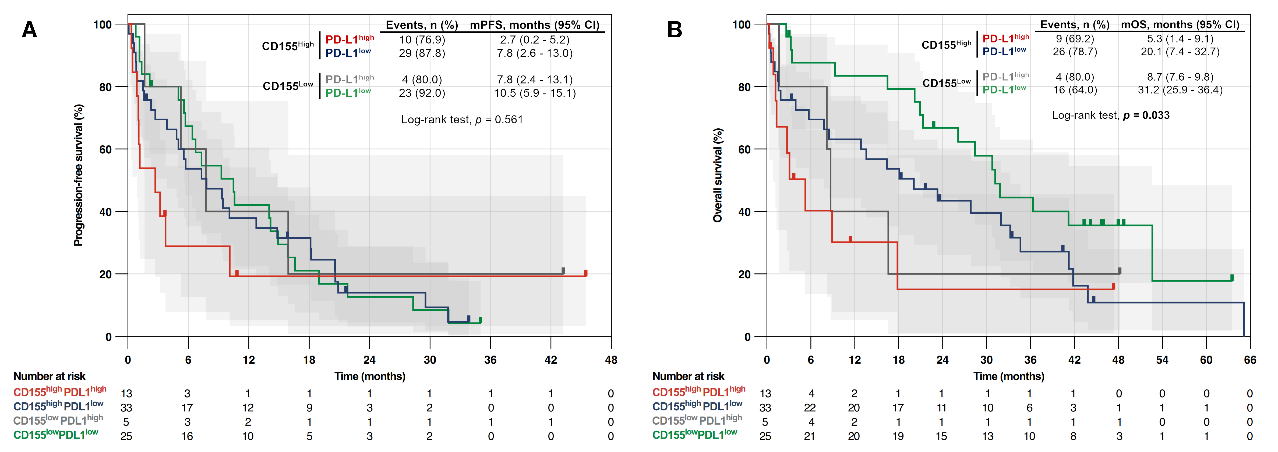


**10. “Moreover, data in the literature show that CD155 expression impairs anti-PD1 therapy response in non-small cell lung cancer. Thus, I suggest the authors apply a decision tree to identify how the markers they included in the manuscript can work together to identify overall survival and progression-free survival.”**

**Response**: a decision tree was designed based on your advice as **Supplementary Figure 5**:


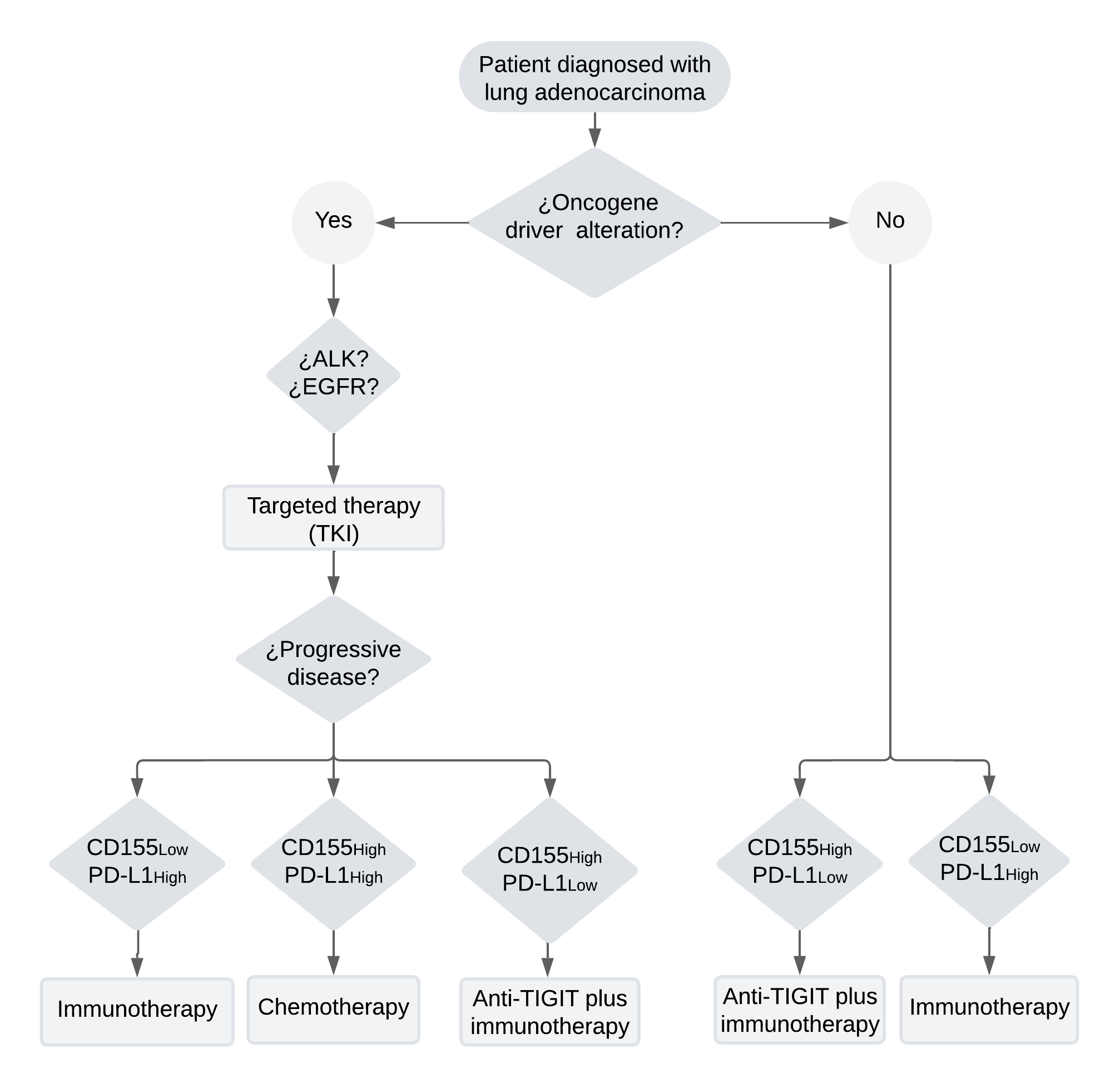


**Supplementary figure 5. Decision tree of therapeutic management of patients with lung adenocarcinoma based on the expression of CD155 and PD-L1.** CD155, cluster of differentiation 155. PD-L1, programmed death ligand 1. PD-L1High refers to a tumor proportion score (TPS) of ≥50%, while PD-L1Low for tumors with a TPS <50%. CD155Low refers to tumors having a TPS <110 for patients negative for driver oncogenic alterations, and CD155High for patients positive for oncogenic driver alterations harboring a TPS ≥155.

REVIEWER #3

**11. “An expanded description of patient cohort selection criteria is essential to address potential biases.”**

**Response:** description of patient’s cohort selection was reformulated as follows (**page 6, lines 132-135**):

“An observational cohort study was conducted on patients with advanced lung cancer diagnosed between January and October 2019 at the Thoracic Oncology Unit of the Instituto Nacional de Cancerología (INCan). Individuals with confirmed diagnosis of lung cancer undergoing at least one line of anticancer therapy and having available histologic tumor samples were eligible for analysis.”

**12. “Further elaboration on the rationale behind the choice of statistical tests and models would enhance the manuscript's analytical framework.”**

**Response:** nature of variables, data distribution and objective outcomes represented the central axis for statistical tests’ choice, and their description was reformulated in methods section (**page 8, lines 180-193**):

“The cut-off values for defining high or low CD155 staining were estimated using receiver operating characteristic (ROC) curves according to 2-year survival for the whole population and subgroups with or without driver oncogenic alterations using GraphPad Prism 9.0.1 for macOS (Dotmatics, California, United States). Continuous variables, including age, packs per year, and PD-L1 TPS were reported as means and standard deviations (SD), or as medians and interquartile ranges (IQR) based on data distribution assessed by Kolmogorov–Smirnov Test. According to data distribution, comparisons for continuous variables between groups were evaluated using the Student’s t-test or Mann–Whitney U-test. Categorical variables, such as high or low CD155 expression and clinicopathological features were reported as frequencies and proportions, and comparisons between them were analyzed by χ2 test or Fisher exact test, based on their distribution. Survival was examined using the Kaplan–Meier method, and the significance of differences was evaluated using a log-rank test. Variable effects on survival time were investigated using the Cox regression model. Statistical significance was set at p<0.05. All statistical analyses were performed using SPSS software (version 19.0; International Business Machines Corporation, Chicago, Illinois, USA).”

**13. “The manuscript lacks a comparative analysis with existing literature on similar biomarkers in NSCLC.” “A discussion regarding how these findings align or contrast with other studies, including any observed discrepancies, would be beneficial.”**

**Response:** a comparative analysis of similar biomarkers was added to the discussion section (**page 12, lines 293-301**):

“Other nectin-like molecules have also demonstrated clinical importance in lung cancer, such as CD112 and CD113, but CD155 raises as the most representative due to its higher affinity for TIGIT, which increases its importance as a potential target of therapeutic inhibition [21]. CD113 is also frequently found in lung adenocarcinoma (25%) and only represents a deleterious prognostic factor when not co-localized with E-cadherin in cell membrane, likely allowing its binding with nectin-5 or TIGIT to promote cancer progression [22]. Otherwise, high serum levels of CD112 correlated with clinical stage, tumor size and metastatic status, but was not considered a significant predictor of progression-free survival in lung cancer [23], probably derived from its weak interaction with TIGIT [24].”

**14. “Discussions on potential confounders that might influence the interpretation of CD155 as an independent prognostic factor, such as various treatment modalities, would provide a more nuanced understanding of the results.”**

**Response:** potential confounders were declared in limitations section **(page 14, lines 331-332)**

“As well, potential confounders of this study are multiple treatment modalities with considerably different impact on survival outcomes and inter-individual variability due to our small-sample size.”

**15. “Minor grammatical corrections are advised to enhance clarity, particularly in the introduction and discussion sections.”**

**Response**: grammatical corrections were made to enhance clarity of our manuscript in introduction and discussion sections.

**16. “An explicit statement regarding patient consent, especially given the study's involvement with patient tissue samples, should be included.”**

**Response:** ethical approval and patients’ informed consent statement was added to methods section **(page 6, lines 141-144**):

“The Institutional Ethics and Scientific Board Review Committee approved this study [(018/063(ICI) (CEI/1303/18)]. All personal data from enrolled patients were kept confidential using an intern number code to identify samples, and not personal data, and thereby, informed consent was not applicable.”

**17. “To further substantiate CD155's role as a biomarker, consider incorporating additional experiments or data analyses, such as its relationship with responses to specific treatments.”**

**Response:** this analysis was performed, and its results are described in section 3.6 as follows (**page 11, lines 259-263**):

“In the entire cohort, 31 patients (36.0%) showed objective response rate (ORR) (Table 1). No significant differences were identified in terms of therapeutic response according to CD155 expression. Nonetheless, a trend to lower ORR to chemotherapy was observed in patients with CD155high (20% vs 37.5%, p=0.307). No important differences were found in patients undergoing targeted therapies for EGFR mutations or ALK rearrangements (**Supplementary Figure 4**).”

And they were presented in discussion section (**page 12, lines 281-286**):

“This study identified trend to worse objective response rate in chemotherapy-treated patients having a CD155High, which is consistent with previous evidence. This has been attributed to its relationship with a solid histologic pattern, which is commonly considered a predictor for poor chemotherapy response [16]. Although previous evidence relates CD155 with lower response to EGFR TKIs in early-stage lung cancer [16], this study did not show important differences in our population. This discrepancy may mostly derive from disease stage and type of TKIs evaluated.”

Their associated Figure was located as **Supplementary Figure 4**:


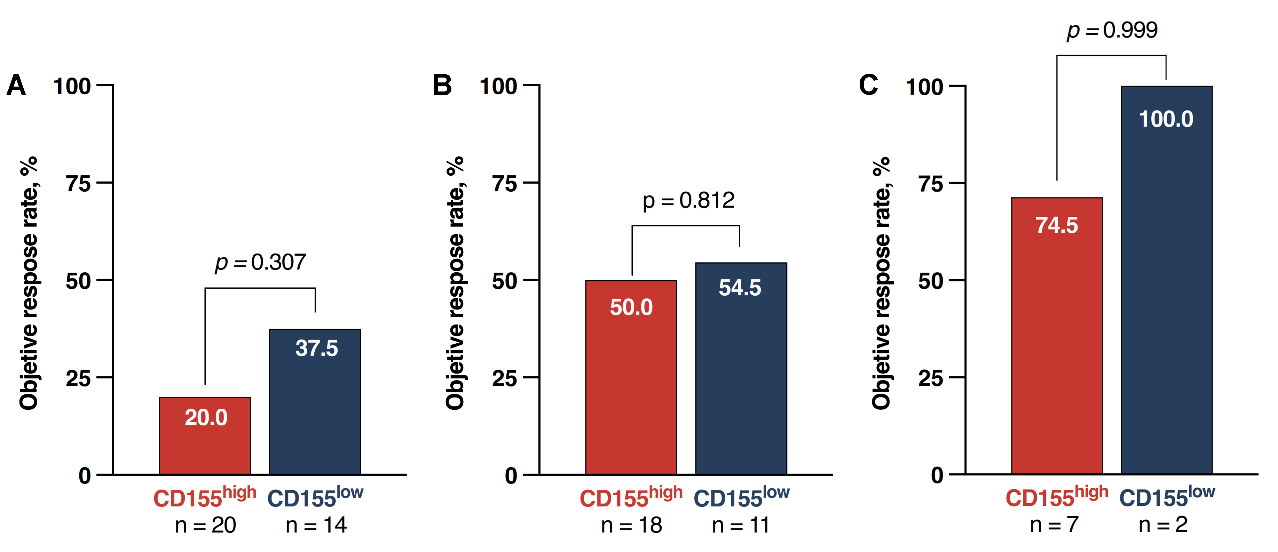


**18. “Ensure all figures and tables are accurately referenced within the text, and their legends comprehensively describe the content.”**

**Response:** all figures and tables are accurately referenced within the text, and their legends are comprehensive.

**19. “The reference list should be updated and expanded to include recent studies investigating the role of CD155 in cancer prognosis.”**

**Response:** the list of references was updated, and they were added recent studies regarding the prognostic and therapeutic role of CD155 axis (highlighted in **bold red color**) (**Page 16**).

“1. Oyama R, Kanayama M, Mori M, Matsumiya H, Taira A, Shinohara S, et al. CD155 expression and its clinical significance in non-small cell lung cancer. Oncol Lett. 2022;23:1–8.

2. Sharma P, Hu-Lieskovan S, Wargo JA, Ribas A. Primary, Adaptive, and Acquired Resistance to Cancer Immunotherapy. Cell. 2017;168:707–23.

3**. Zhang D, Liu J, Zheng M, Meng C, Liao J. Prognostic and clinicopathological significance of CD155 expression in cancer patients: a meta-analysis. World J Surg Oncol [Internet]. 2022;20. Available from: https://doi.org/10.1186/s12957-022-02813-w**

**4. Zhang H, Liu Q, Lei Y, Zhou J, Jiang W, Cui Y, et al. Direct interaction between CD155 and CD96 promotes immunosuppression in lung adenocarcinoma. Cell Mol Immunol [Internet]. 2021;18:1575–7. Available from: http://dx.doi.org/10.1038/s41423-020-00538-y**

5. O’Donnell JS, Madore J, Li XY, Smyth MJ. Tumor intrinsic and extrinsic immune functions of CD155. Semin Cancer Biol [Internet]. 2020;65:189–96. Available from: https://doi.org/10.1016/j.semcancer.2019.11.013

6. Lepletier A, Madore J, O’Donnell JS, Johnston RL, Li XY, McDonald E, et al. Tumor CD155 Expression Is Associated with Resistance to Anti-PD1 Immunotherapy in Metastatic Melanoma. Clinical Cancer Research. 2020;26:3671–81.

**7. Jiang C, Qu X, Ma L, Yi L, Cheng X, Gao X, et al. CD155 expression impairs anti-PD1 therapy response in non-small cell lung cancer. Clin Exp Immunol. 2022;208:220–32.**

8. Nakai R, Maniwa Y, Tanaka Y, Nishio W, Yoshimura M, Okita Y, et al. Overexpression of Necl-5 correlates with unfavorable prognosis in patients with lung adenocarcinoma. Cancer Sci. 2010;101:1326–30.

9. Xu Y, Cui G, Jiang Z, Li N, Zhang X. Survival analysis with regard to PD-L1 and CD155 expression in human small cell lung cancer and a comparison with associated receptors. Oncol Lett. 2019;17:2960–8.

10. Kono T, Imai Y, Yasuda SI, Ohmori K, Fukui H, Ichikawa K, et al. The CD155/poliovirus receptor enhances the proliferation of ras-mutated cells. Int J Cancer. 2008;122:317–24.

11. Capdeville C, Russo L, Penton D, Migliavacca J, Zecevic M, Gries A, et al. Spatial proteomics finds CD155 and Endophilin-A1 as mediators of growth and invasion in medulloblastoma. Life Sci Alliance. 2022;5.

12. Avilés-Salas A, Flores-Estrada D, Lara-Mejía L, Catalán R, Cruz-Rico G, Orozco-Morales M, et al. Modifying factors of PD-L1 expression on tumor cells in advanced non-small-cell lung cancer. Thorac Cancer. 2022;13:3362–73.

13. Sho M, Nishiwada S, Yasuda S, Shimada K, Yamato I, Akahori T, et al. Clinical Significance of CD155 Expression in Human Pancreatic Cancer. Anticancer Res [Internet]. 2015 [cited 2023 Nov 24];35:2287–97. Available from: https://pubmed.ncbi.nlm.nih.gov/25862891/

14. Kakunaga S, Ikeda W, Shingai T, Fujito T, Yamada A, Minami Y, et al. Enhancement of serum- and platelet-derived growth factor-induced cell proliferation by Necl-5/Tage4/poliovirus receptor/CD155 through the Ras-Raf-MEK-ERK signaling. Journal of Biological Chemistry. 2004;279:36419–25.

15. Gao J, Zheng Q, Shao Y, Wang W, Zhao C. CD155 downregulation synergizes with adriamycin to induce breast cancer cell apoptosis. Apoptosis. 2018;23:512–20.

16. Zhang Y, Li J, Wang R, Li Y, Pan Y, Cai D, et al. The prognostic and predictive value of solid subtype in invasive lung adenocarcinoma. Sci Rep. 2014;4:1–6.

17. Makinoshima H, Takita M, Matsumoto S, Yagishita A, Owada S, Esumi H, et al. Epidermal growth factor receptor (EGFR) signaling regulates global metabolic pathways in EGFR-mutated lung adenocarcinoma. Journal of Biological Chemistry. 2014;289:20813–23.

18. Li YC, Zhou Q, Song QK, Wang R Bin, Lyu S, Guan X, et al. Overexpression of an Immune Checkpoint (CD155) in Breast Cancer Associated with Prognostic Significance and Exhausted Tumor-Infiltrating Lymphocytes: A Cohort Study. J Immunol Res. 2020;2020.

19. Ikeda J, Ohe C, Yoshida T, Saito R, Tsuta K, Kinoshita H. CD155 immunohistochemical expression in upper tract urothelial carcinoma predicts poor prognosis. Oncol Lett. 2022;24.

20. Lepletier A, Madore J, O’Donnell JS, Johnston RL, Li XY, McDonald E, et al. Tumor CD155 Expression Is Associated with Resistance to Anti-PD1 Immunotherapy in Metastatic Melanoma. Clinical Cancer Research. 2020;26:3671–81.

21. Chan CJ, Andrews DM, Smyth MJ. Receptors that interact with nectin and nectin-like proteins in the immunosurveillance and immunotherapy of cancer. Curr Opin Immunol. 2012;24:246–51.

22. Maniwa Y, Nishio W, Okita Y, Yoshimura M. Expression of nectin 3: Novel prognostic marker of lung adenocarcinoma. Thorac Cancer. 2012;3:175–81.

**23. Erturk K, Karaman S, Dagoglu N, Serilmez M, Duranyildiz D, Tas F. Serum nectin-2 and nectin-4 are diagnostic in lung cancer: which is superior? Wien Klin Wochenschr. 2019;131:419–26.**

24. Yu X, Harden K, C Gonzalez L, Francesco M, Chiang E, Irving B, et al. The surface protein TIGIT suppresses T cell activation by promoting the generation of mature immunoregulatory dendritic cells. Nat Immunol. 2009;10:48–57.

25. Gong K, Guo G, Panchani N, Bender ME, Gerber DE, Minna JD, et al. EGFR inhibition triggers an adaptive response by co-opting antiviral signaling pathways in lung cancer. Nat Cancer. 2020;1:394–409.

26. Heo JY, Park C, Keam B, Ock CY, Kim M, Kim TM, et al. The efficacy of immune checkpoint inhibitors in anaplastic lymphoma kinase-positive non-small cell lung cancer. Thorac Cancer. 2019;10:2117–23.

27. Oyama R, Kanayama M, Mori M, Matsumiya H, Taira A, Shinohara S, et al. CD155 expression and its clinical significance in non small cell lung cancer. Oncol Lett. 2022;23:166.

**28. Kojima K, Sakamoto T, Kasai T, Kagawa T, Yoon H, Atagi S. PD-L1 expression as a predictor of postoperative recurrence and the association between the PD-L1 expression and EGFR mutations in NSCLC. Sci Rep. 2021;11.**

**29. Cho BC, Abreu DR, Hussein M, Cobo M, Patel AJ, Secen N, et al. Tiragolumab plus atezolizumab versus placebo plus atezolizumab as a first-line treatment for PD-L1-selected non-small-cell lung cancer (CITYSCAPE): primary and follow-up analyses of a randomised, double-blind, phase 2 study. Lancet Oncol. 2022;23:781–92.**

**30. Rousseau A, Parisi C, Barlesi F. Anti-TIGIT therapies for solid tumors: a systematic review. ESMO Open. 2023;8:101184**.”
