# Supplementary material for: Prognostic impact of nectin-like molecule-5 (CD155) expression in non-small cell lung cancer
Source: J Transl Med. 2024 Sep 12;22:841. doi: 10.1186/s12967-024-05471-6 (PMC11391680; doi:10.1186/s12967-024-05471-6)
Supplement: Supplementary file 3 — Supplementary Material 3 [file 12967_2024_5471_MOESM3_ESM.docx]

**Prognostic impact of nectin-like molecule-5 (CD155) expression in Non-Small Cell Lung Cancer**

Popa-Navarro Xitlally^3^, Avilés-Salas Alejandro^2^, Hernández-Pedro Norma^3^*, Orozco-Morales Mario^3^, Caballé-Pérez Enrique^1,3^, Castillo-Ruiz Cesar^3^, Lucio-Lozada José^3^, Barrios-Bernal Pedro^3^, Hernandez-Martinez Juan-Manuel^3,4^, Arrieta Oscar^1,3,^*.

^1^Thoracic Oncology Unit, Instituto Nacional de Cancerología (INCan), Mexico City 14080, Mexico.

^2^Pathology department, Instituto Nacional de Cancerología (INCan), Mexico City 14080, Mexico.

^3^Personalized Medicine Laboratory, Instituto Nacional de Cancerología (INCan), Mexico City 14080, Mexico.

^4^CONAHCYT-Instituto Nacional de Cancerología, Mexico City, Mexico

* Correspondence: [ogar@unam.mx](mailto:ogar@unam.mx); [nhernandezp@incan.edu.mx](mailto:nhernandezp@incan.edu.mx)

**HIGHLIGHTS**

- Among patients with EGFR alterations, CD155 expression was higher in exon 19 deletion than L858R mutation.
- High CD155 expression was associated with higher PD-L1 positivity (TPS >1%) among individuals with *ALK* or *EGFR* alterations.
- High CD155 expression is a predictor of worse survival outcomes in patients with advanced NSCLC predominantly among those without onco-driver mutations.
