# Supplementary material for: Prognostic impact of nectin-like molecule-5 (CD155) expression in non-small cell lung cancer
Source: J Transl Med. 2024 Sep 12;22:841. doi: 10.1186/s12967-024-05471-6 (PMC11391680; doi:10.1186/s12967-024-05471-6)
Supplement: Supplementary file 4 — Supplementary Material 4 [file 12967_2024_5471_MOESM4_ESM.docx]

Oscar Arrieta reports receiving personal fees from Pfizer, Lilly, Merck, and Bristol-Myers Squibb and grants and personal fees from AstraZeneca, Boehringer Ingelheim, and Roche, outside of this submitted work. The rest of authors declare no affiliations with or involvement in any organization or entity with any financial interest in the subject matter or materials discussed in this manuscript.
