# Supplementary material for: Prognostic impact of nectin-like molecule-5 (CD155) expression in non-small cell lung cancer
Source: J Transl Med. 2024 Sep 12;22:841. doi: 10.1186/s12967-024-05471-6 (PMC11391680; doi:10.1186/s12967-024-05471-6)

Supplementary figure 1


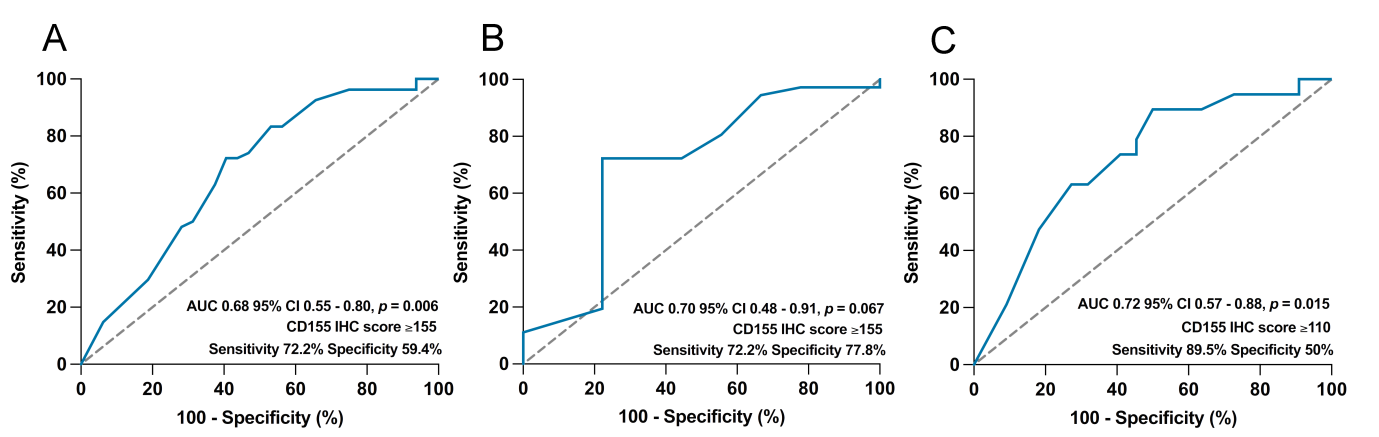


Supplementary figure 2


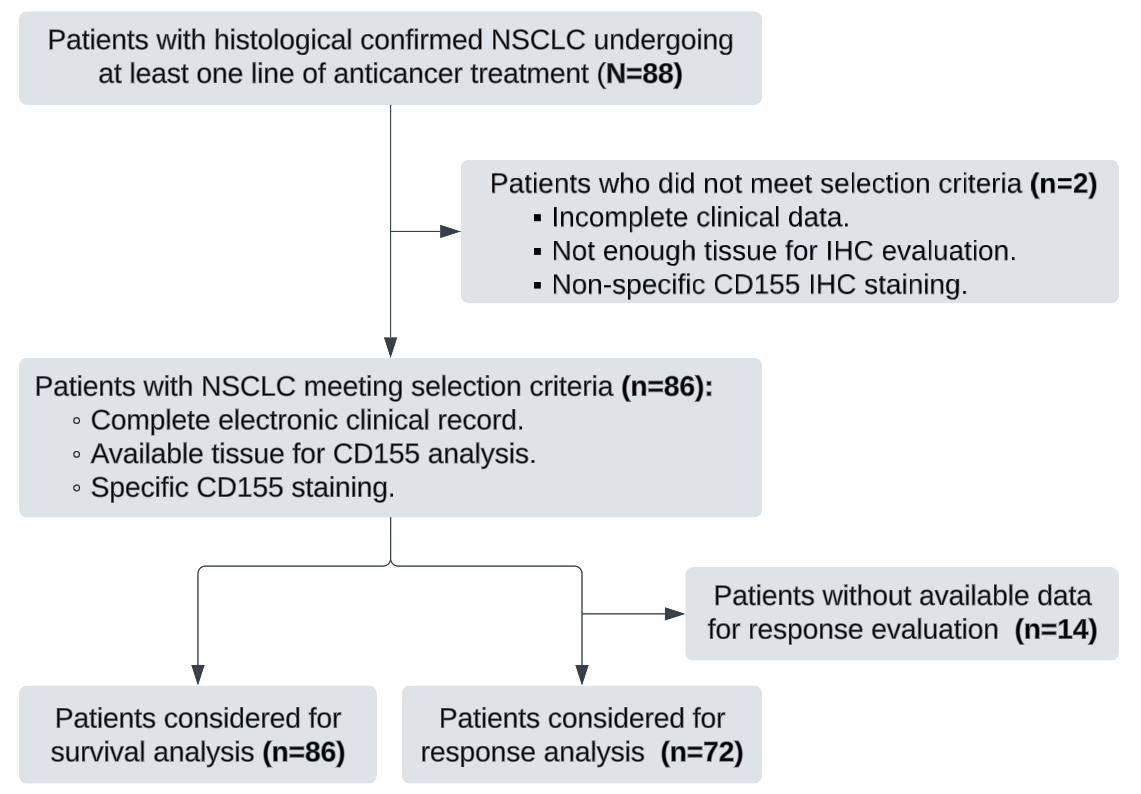


Supplementary figure 3


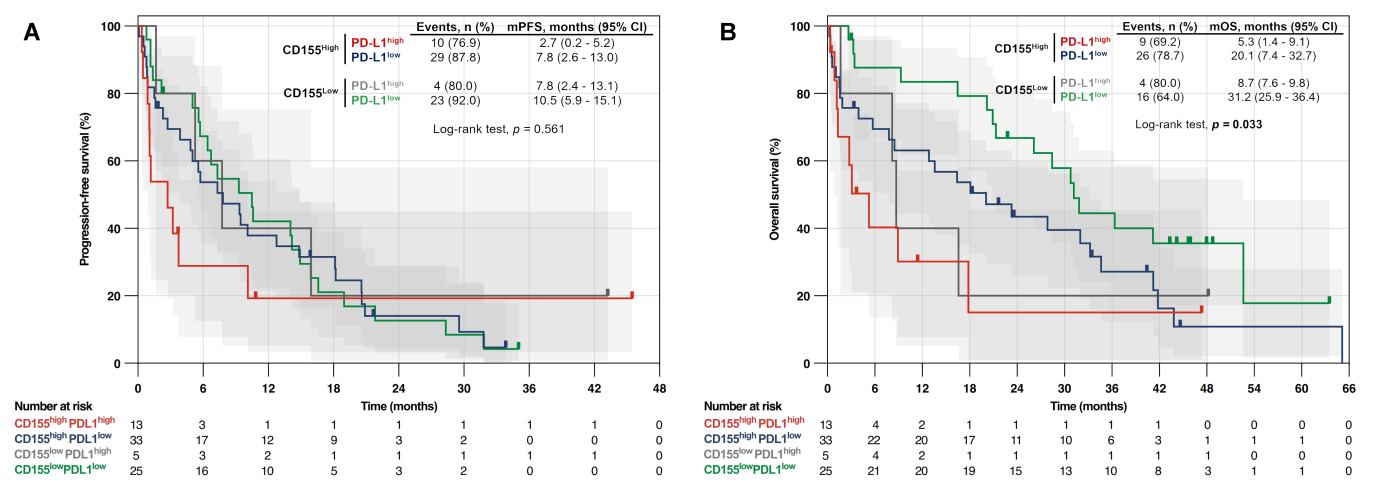


Supplementary figure 4


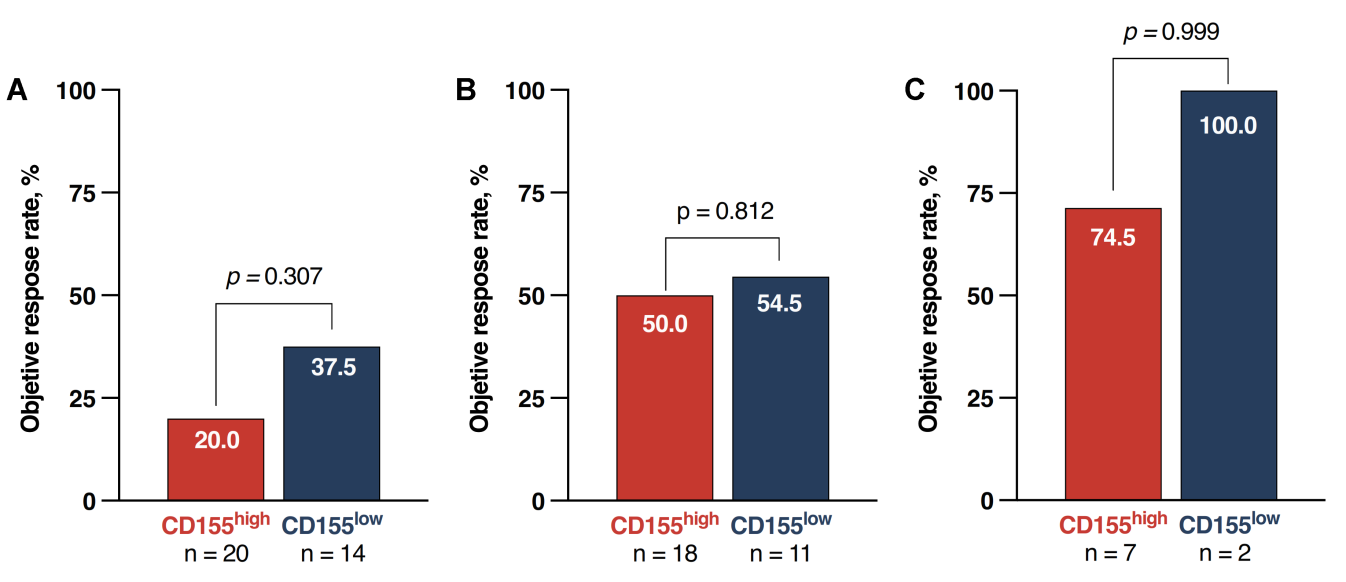


Supplementary figure 5


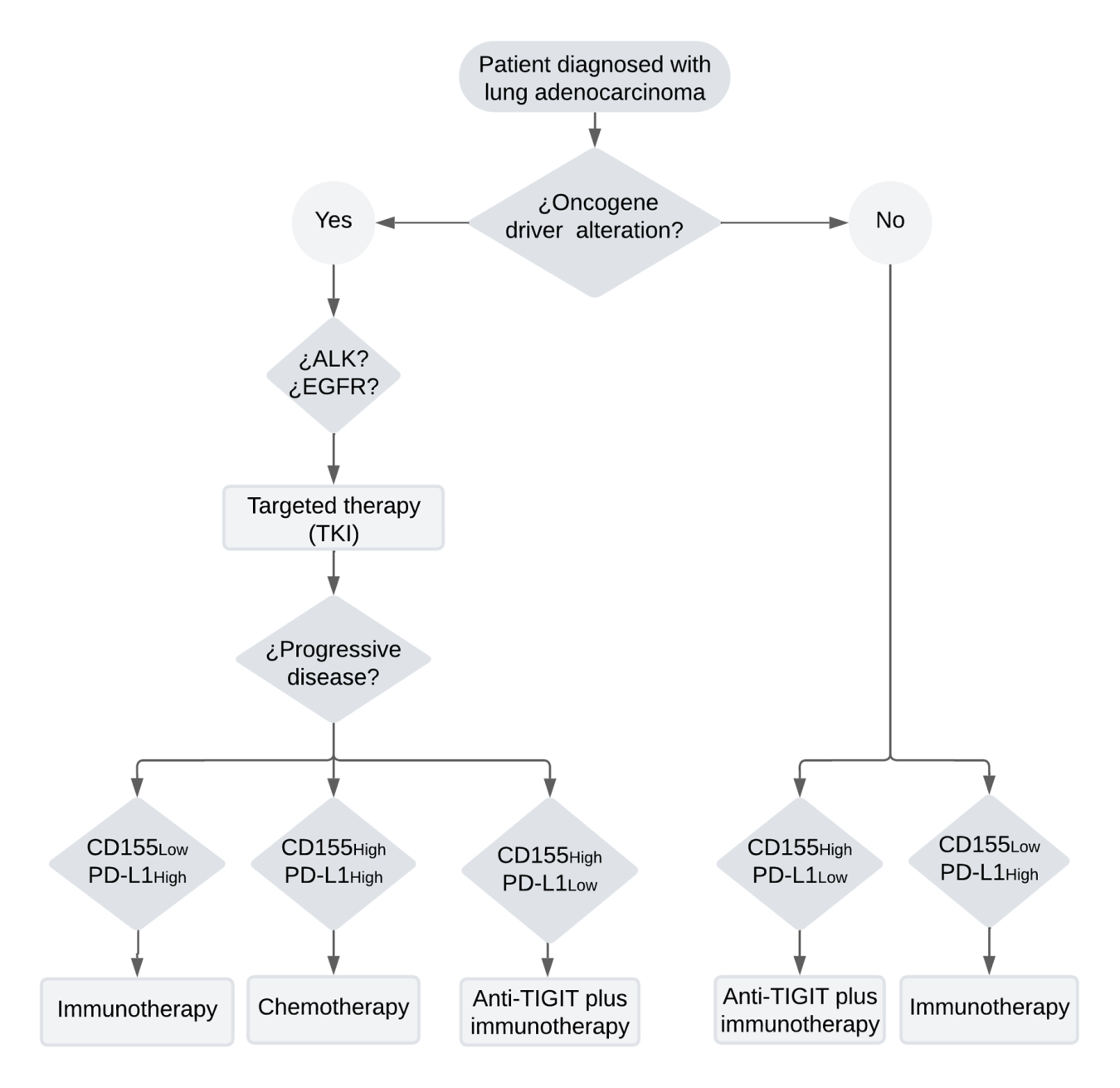

Supplement: Supplementary file 5 — Supplementary Material 5 [file 12967_2024_5471_MOESM5_ESM.docx]
